# Hepatic methotrexate-associated lymphoproliferative disease: a case report and literature review

**DOI:** 10.1186/s40792-024-02065-8

**Published:** 2024-11-15

**Authors:** Shinya Sakamoto, Motoyasu Tabuchi, Rika Yoshimatsu, Manabu Matsumoto, Jun Iwata, Takehiro Okabayashi

**Affiliations:** 1 Department of Gastroenterological Surgery, Kochi Health Sciences Center, 2125-1 Ike, Kochi-City, Kochi 781-8555 Japan; 2 Department of Radiology, Kochi Health Sciences Center, 2125-1 Ike, Kochi-City, Kochi 781-8555 Japan; 3 Department of Diagnostic Pathology, Kochi Health Sciences Center, 2125-1 Ike, Kochi-City, Kochi 781-8555 Japan

**Keywords:** Methotrexate, Methotrexate-associated lymphoproliferative disease, Liver surgery

## Abstract

**Background:**

Methotrexate-associated lymphoproliferative disease (MTX–LPD) is a rare and life-threatening complication of MTX administration. MTX–LPD features more extranodal lesions than malignant lymphoma; however, the liver is an extremely rare organ that develops LPD. Herein, we present a case of hepatic MTX–LPD treated with surgical resection. We also reviewed the literature on hepatic MTX–LPD.

**Case presentation:**

A 66-year-old man with a history of rheumatoid arthritis (RA) was admitted to our department for the treatment of hepatic solitary liver tumor. The patient had been receiving MTX (14 mg/week) for RA for 6 years. MTX was withdrawn and salazosulfapyridine was prescribed 3 weeks prior to admission because of mediastinal MTX–LPD. Abdominal contrast-enhanced computed tomography showed a slightly ring-like enhanced hypovascularized mass (80 mm) in the lateral section of the liver. Carbohydrate antigen 19-9 (78.1 U/mL) level was elevated. No evidence was observed on esophagogastroduodenoscopy or colonoscopy. The tumor was suspected to be an intrahepatic cholangiocarcinoma. The patient underwent hepatic lateral sectionectomy and lymphadenectomy. Pathological examination revealed that the hepatic mass was coagulative necrosis of the CD20-positive B-cell lymphocytes. These histological findings were similar to those of rapid necrotic lymphoma. MTX–LPD is known to spontaneously regress after withdrawing MTX, and the patient was diagnosed with hepatic MTX–LPD.

**Conclusions:**

MTX–LPD can occur in the liver. Clinician should suspect hepatic MTX–LPD when a liver mass is detected in patient who had been treating with MTX for RA.

## Background

Methotrexate (MTX) is an immunosuppressant commonly used to treat rheumatoid arthritis (RA), which rarely leads to lymphoproliferative disease (LPD) [1]. When occurs, it is termed MTX-associated LPD (MTX–LPD). Forty-to-seventy percent of patients with MTX–LPD have extranodal lesions, including those in the lung, skin, oral cavity, and spleen [2]. However, hepatic MTX–LPD is extremely rare and its clinical features are not well known. Herein, we present a case of hepatic MTX–LPD treated with surgical resection. We also reviewed the literature on hepatic MTX–LPD.

## Case presentation

**Fig. 1:**
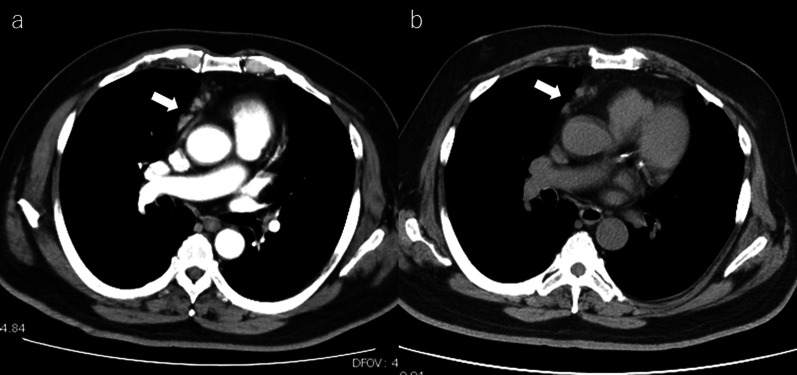
**a** Computed tomography (CT) showed slightly enlargement of mediastinal lymph nodes at the diagnosis. **b** CT showed no change in size 1 month later after withdrawing MTX

A 66-year-old man with a solitary liver tumor was referred to our department. The patient had a history of RA for which MTX (14 mg/week) was administered. He also had a medical history of essential hypertension, asthma, and dyslipidemia. Computed tomography (CT) was performed because of elevated C-reactive protein (CRP) levels at another hospital. CT revealed enlargement of the mediastinal lymph nodes and a solitary liver mass of 8 cm in diameter (Fig. 1a). The mediastinal lymph node was suspected to be MTX–LPD, which was also the cause of the increased inflammatory response. The patient’s family physician discontinued MTX and prescribed salazosulfapyridine, and the patient was referred to our hospital for further examination and treatment of the liver tumor. The patient was prescribed antihypertensive drugs, inhaled corticosteroids, and long-acting β-agonists. He had no history of hepatitis or alcohol consumption. He was obese, with a body mass index of 34.9. White blood cell count (12,600/μL) and CRP (8.03 mg/dL), lactate dehydrogenase (365 U/mL), and carbohydrate antigen 19-9 (78.1 U/mL) levels were elevated. Circulating liver enzymes and serum bilirubin levels were within normal limits. Alpha-fetoprotein (6.9 ng/mL), protein induced by vitamin K absence or antagonist-II (7 mAU/mL), and carcinoembryonic antigen (3.1 ng/mL) levels were not elevated.

**Fig. 2:**
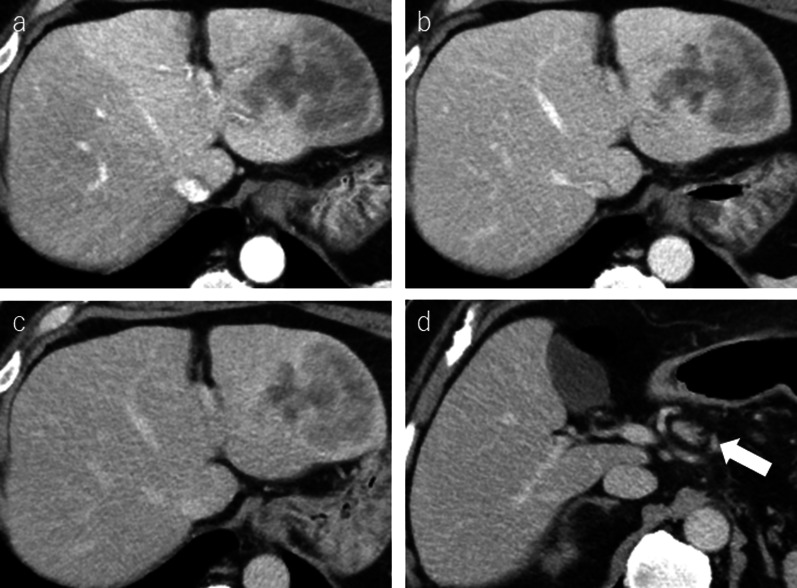
**a**–**c** Contrast-enhanced computed tomography shows an 80 mm heterogeneous hypovascular mass in the lateral section. The mass is gradually enhanced from early to late phase. **d** Lymph node of anterosuperior common hepatic artery is enlarged, suspicious of metastasis (arrow)

**Fig. 3:**
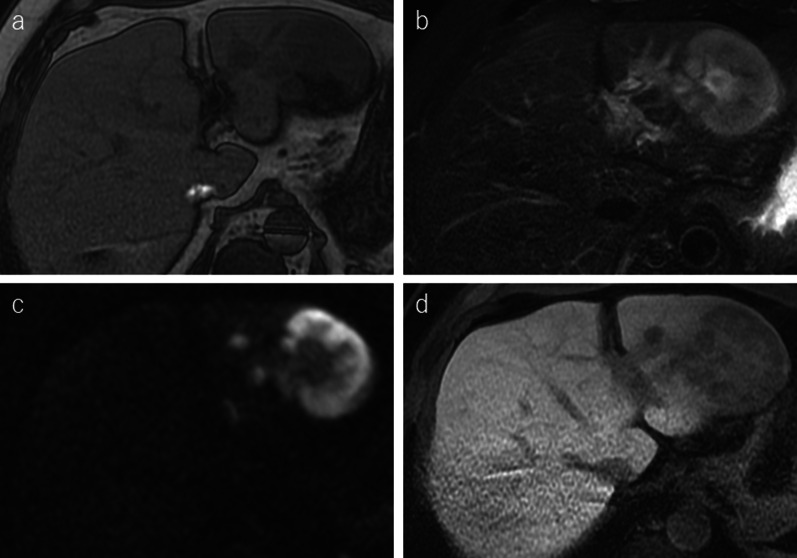
Magnetic resonance imaging (MRI) shows low intensity of the mass on T1-weighted images (**a**), high intensity on T2-weighted images (**b**), high intensity on diffusion-weighted images (**c**), and low intensity in the hepatobiliary phase of gadoxetic acid-enhanced MRI

Abdominal contrast-enhanced CT showed a slightly ring-like enhanced hypovascularized mass of 80 mm in the lateral section of the liver (Fig. 2a–c). The intra-abdominal lymph nodes in the lesser omentum (#3) and around the common hepatic arteries (#8a) were also enlarged (Fig. 2d). Magnetic resonance imaging (MRI) revealed that the mass had low intensity on T1-weighted images, heterogeneous high intensity on T2-weighted images, and high intensity at the tumor edge on diffuse-weighted images. Gd-EOB–DAPT enhancement MRI showed diminishment of Gd-EOB–DAPT on the hepatobiliary phase (Fig. 3). Esophagogastroduodenoscopy and colonoscopy did not reveal gastrointestinal tract lesions. Therefore, intrahepatic cholangiocarcinoma was suspected. Surgical resection was planned 3 weeks after MTX withdrawal. A lateral sectionectomy and lymphadenectomy were performed. The patient’s postoperative course was uneventful and was discharged on postoperative day 7 without any complications.

**Fig. 4:**
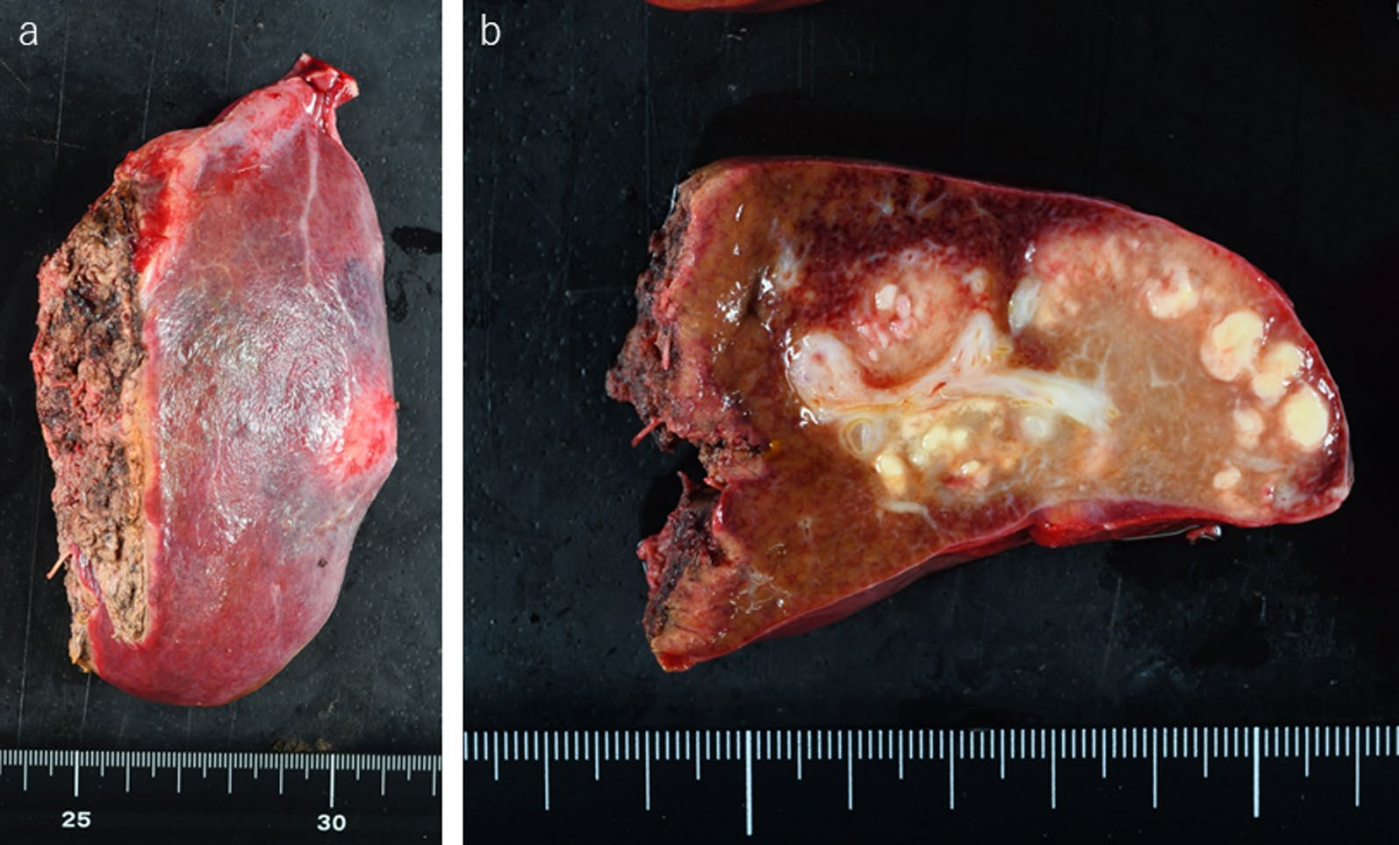
**a** Resected specimen after lateral sectionectomy and cholecystectomy. **b** Light brown lesion with scattered white nodule-like area with irregular borders is shown in the resected specimen

**Fig. 5:**
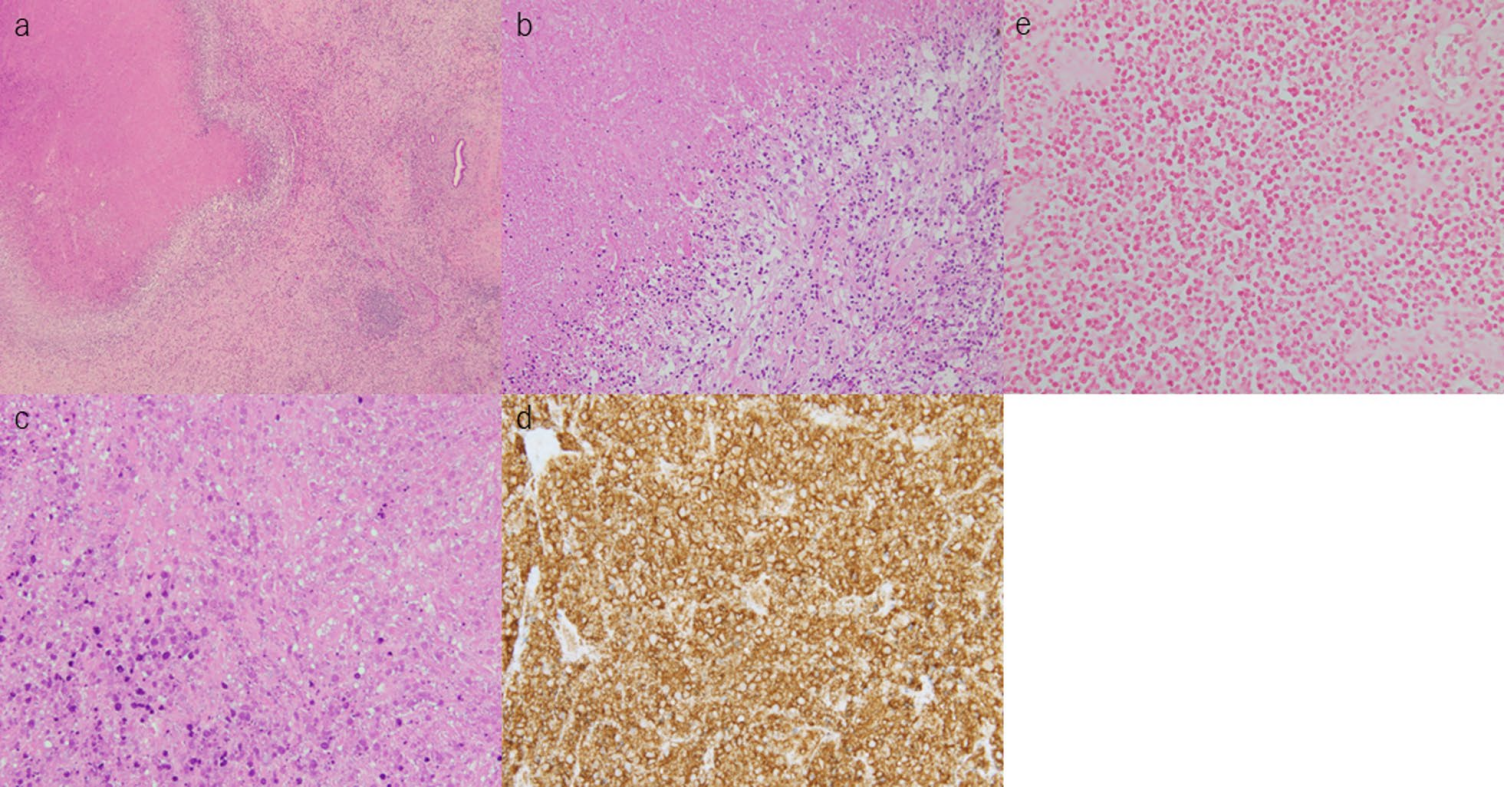
**a**–**c** Coagulative necrosis of small round cells surrounded by macrophages and lymphocytes is revealed. No viable atypical lymphocytes are found. **a** Hematoxylin–eosin [H&E] stain; ×4, **b** H&E stain; ×10, **c** H&E stain; ×40, **d** CD20 is positive for necrotic lymphocytes in immunohistological examination (CD 20 stain; ×40). **e** EBV-encoded small RNA (EBER) is negative for necrotic lymphocytes. (EBER stain; ×40)

Grossly, a light-brown lesion with scattered white nodule-like areas and irregular borders was observed in the resected specimen (Fig. 4). Histological examination revealed coagulative necrosis of small round cells surrounded by macrophages and lymphocytes (Fig. 5a–c). No viable atypical lymphocytes were detected. Immunohistological examination revealed that the necrotic cells were CD20-positive B-cell lymphocytes (Fig. 5d). Although EBV-encoded small RNA (EBER) was negative (Fig. 5e), these findings, similar to those observed in rapid necrotic lymphomas, and high serum lactate dehydrogenase levels, enlarged mediastinal lymph nodes, and history of MTX withdrawal 3 weeks prior to admission led to the diagnosis of hepatic MTX–LPD. Pathological examination revealed the lymph node preoperatively detected by CT scan was normal. He discharged our hospital on postoperative day 7 without any complication. The mediastinal lymph nodes were no change in size 1 month later after MTX discontinuation (Fig. 1b). At the follow-up examination in the outpatient clinic 9 months postoperatively, no evidence of recurrence was detected.

## Discussion

MTX is a key drug used in the management RA worldwide. MTX–LPD is a rare, but well-known, life-threatening complication that develops in patients with RA treated with MTX [3]. Because MTX is an anti-folate metabolic agent, its action on the folate metabolic pathway may affect the risk of LPD development [4]. Approximately 60% of patients with MTX–PLD are Epstein–Barr virus (EBV) positive. Since MTX reactivates the latent EBV owing to its immunosuppressive effects, mechanism for the development of MTX–LPD via EBV reactivation has been reported [5]. MTX–LPD has a generally favorable prognostic outcome compared to lymphoma [6]. Spontaneous regression is seen in 50–70% of MTX–LPD cases after MTX withdrawal [6]. Moreover, EBER positivity was considered to be a favorable sign of regression [3].

MTX–LPD frequently involve extranodal lesions, whereas hepatic MTX–LPD is extremely rare [2]. To the best of our knowledge, only 17 cases, including ours, have been reported in the English literature (Table 1). Of this, 11 patients had multiple lesions compared to five with single lesions. Four patients had lesions outside the liver, including in the lungs, spleen, abdominal para-aortic lymph nodes, adrenal glands, and mediastinal lymph nodes. In the current case, enlargement of mediastinal lymph nodes was clue of diagnosed with MTX–LPD; however, the lymph nodes were small and round shape, and showed only little change after MTX-discontinuation. One previous report described a case with mediastinal lymphadenectomy [8]. Because the appearance of mediastinal lymph nodes and those clinical courses were different between previous case and our case, the mediastinal lymph nodes might not be a true lesion of MTX–LPD in the current case. The incidence of hepatic MTX–LPD increases with age, and 76% of patients were aged > 60 years. The median age of the patients was 64 years, and the prevalence was more in female patients (56%). The background disease was almost RA except for one adult-onset Still’s disease. The duration of MTX administration ranged from 5 months to 17 years. The median MTX administration period was 6 years. Fever, malaise, and abdominal pain were common symptoms; however, approximately 40% (7/17) of patients, including our patient, were asymptomatic.

**Table 1:** Summary of 17 hepatic MTX–LPD cases published in English literature including our case

No	Ref.	Age/sex	Primary disease	Number of tumors	Other lesions	Dose of MTX [/week]	Duration of MTX administration	Symptom	Contrast CT findings	Pathology	Treatment	Outcome	Prognosis/follow-up time
1	[25]	48/F	RA	Multiple	None	7.5 mg	15 months	None	Ring-like enchantment	DLBCL	Discontinued	Disappear in 3 months	Alive/2 years
2	[18]	69/M	RA	Solitary	None		Unknown	None	Slightly enhancement	Lymphocytic infiltrates	Discontinued	Disappear in 3 months	Alive/3 months
3	[19]	64/M	RA	Multiple	None	6 mg	6 years	Fever, RA	Poor enhancement with irregular margin	DLBCL	Chemotherapy	CR in 5 months	Alive/1 years
4	[7]	56/F	RA	Multiple	None	6–16 mg	6 years	Malaise, weight loss, fever	Poor enhancement with irregular margin PV was patent in the mass	DLBCL	Discontinued (no change in 3 weeks) → chemotherapy	CR in 6 months	Alive/6 months
5	[13]	59/F	RA	Multiple	None		Unknown	None	Ring-like enchantment	DLBCL	Discontinued	Disappear in 6 months	Alive/6 months
6	[26]	64/M	RA	Multiple	None	8–14 mg	2 years	Malaise, fever, RAQP	Ring-like enchantment	DLBCL	Discontinued (regress in 3 months) → chemotherapy	CR in 8 months	Alive/2 years
7	[9]	63/M	RA	Multiple	None	4–10 mg	10 years	Hepatic dysfunction	No contrast CT	DLBCL	Discontinued	Decreased in size in 3 months	Alive/7 months
8	[15]	63/F	RA	Solitary	None		7 years	None	Slightly enhancement	DLBCL	Hepatectomy	R0 resection No recurrence	Alive/1 years
9	[7]	76/F	RA	Two	Right renal, mediastinum ln		5 years	Fever, abdominal pain	Heterogeneous enhancement, duct penetration sign	B cell	TACE → discontinued	Disappear in 129 days	Alive/129 days
10	[10]	73/F	RA	Solitary	None	1.25–12.5 mg	8 years	Liver enzyme levels were elevated	Slightly enhancement, duct penetration sign	B cell	Discontinued (no change in 3 weeks) → chemotherapy	CR in 3 months	Alive/2 years
11	[27]	82/M	RA	Multiple	Lung, spleen, para-aortic ln	12 mg	9 years	Fever	Poor enhancement with irregular margin	B cell	Discontinued	Disappear in 3 months	Alive/6 months
12	[12]	70/F	RA	n/a	Spleen	5 mg	5 years	Fever, abdominal pain	Poor enhancement	T cell	Discontinued	Disappear in 2 months	Alive/2 years
13	[11]	70/F	RA	Multiple	None		17 years	None	Poor enhancement	DLBCL	TACE → discontinued	Decreased in size in 1 week Disappear in 10 month	Alive/10 months
14	[16]	64/M	RA	Two	None	8–12.5 mg	Unknown	Abdominal pain	Poor enhancement with irregular margin	B cell	Hepatectomy	R0 resection No recurrence	Alive/2 years
15	[14]	57/F	RA	Multiple	Lung		Unknown	Right-sided chest pain	Ring-like enchantment	B cell	Discontinued	Decreased in size in 3 months	Alive/17 months
16	[17]	71/F	AOSD	Solitary	None	6 mg	5 months	None	Poor enhancement	B cell	Discontinued (progress in 7 months) → chemotherapy	PR in 6 months	Alive/6 months
17	Ours	62/M	RA	Solitary	Mediastinum ln	14 mg	6 years	None	Ring-like enchantment	B cell	Hepatectomy	R0 resection No recurrence	Alive/9 months

*RA* rheumatoid arthritis, *DLBCL* diffuse large B cell lymphoma, *RAQP* right upper quadrant pain, *CR* complete response, *PV* portal vein, *PR* partial response, *LN* lymph node, *AOSD* adult onset still disease

Preoperative diagnosis is challenging due to the rarity of hepatic MTX–LPD. No specific imaging findings is not identified for this condition. Generally, hypoechoic lesions are the most common ultrasonographic imaging findings of MTX–LPD in the liver [8–12]. Contrast-enhanced CT generally reveals a low-density mass [8, 12], sometimes with a ring-like enhancement [13, 14]. The masses sometimes exhibit duct-penetrating signs [7, 10]. MRI shows low intensity on T1-weighted images, high intensity on T2-weighted images, and high intensity on diffuse-weighted images [10–12, 15–17]. Gd-EOB–DAPT enhancement MRI reveals that the lesion showed diminished uptake of Gd-EOB–DAPT on the hepatobiliary phase [13]. In the current case, contrast-enhanced CT showed heterogenous poorly enhanced mass, and Gd-EOB–DAPT enhancement MRI showed diminishment of Gd-EOB–DAPT on the hepatobiliary phase. These findings were similar to previous reports. 18F-fluorodeoxyglucose positron emission tomography/computed tomography (FDG–PET/CT) reveals an abnormal accumulation of FDG throughout the tumor [9, 10, 13, 18] or at the tumor edge [16, 17, 19]. And The difference in tumor enhancement or FDG accumulation seemed to reflect the difference between the variable and necrotic lesions. MTX–LPD has a strong tendency towards necrosis, which often occurs internally. MTX–LPD with necrotic changes reveals ring-like enhancement and accumulation of FDG at the tumor edge, whereas MTX–LPD without necrotic changes shows homogenous, slightly enhanced, and strong accumulated FDG in the whole tumor.

These imaging features were generally similar to primary hepatic lymphoma [12, 20]. Duct-penetration sign was known as a clue finding of hepatic lymphoma [20]. The presence of duct-penetrating sign was described in the previous report [7, 10]. We could not investigate the presence of vascular invasion due to no viable tumor cell in our resected specimen, and the other previous reports describing surgically treated cases did not mention the vascular invasion of MTX–LPD. Although the tumor characteristics which is the absence of vascular invasion did not be investigated, duct-penetration sign may be an important imaging finding of MTX–LPD as well as primary hepatic lymphomas.

Pathologic findings were consistent with diffuse large B-cell lymphoma in nine cases, B-cell lymphoma in six, T- and B-cell lymphoma in one, and lymphocytes with interstitial fibrosis in one. Hepatic MTX–LPD are generally B-cell lymphomas; however, T-cell lymphomas have also been reported. EBER was positive in nine reported cases (56%). In the present case, no atypical cells were identified. And EBER was negative. The majority of tumors were necrotic CD 20 positive cells. Our patient underwent surgical resection 3 weeks after MTX discontinuation. MTX–LPD often shows spontaneous regression after MTX withdrawal [6], and the historical findings are similar to those of rapidly necrotic lymphoma. Thus, the patient was diagnosed with MTX–LPD of the liver.

Tissue sampling is extremely important for diagnosis, and liver biopsy is relatively easy to perform. Many cases are diagnosed using biopsy specimens. Patients who have undergone hepatectomy because of the suspicion of a primary malignant tumor in the liver, as in our case, have also been reported [15, 16]. Among the patients diagnosed by biopsy specimens, seven patients showed spontaneous regression after MTX withdrawal, five patients underwent chemotherapy, and four achieved a complete response. Three patients underwent a surgical resection. Two patients underwent transhepatic arterial chemoembolization.

Of the patients with MTX–LPD regardless of the primary location, 62.5% showed at least partial regression of LPD in response to MTX withdrawal without additional antitumor therapy [21]*.* The association between EBV positivity and tumor remission after MTX withdrawal was known. It has been reported that approximately 60% of EBV-positive cases achieved complete remission within 1 month by discontinuing MTX alone, while approximately 30% of EBV-negative cases achieved complete remission within 1 month [1]. Regarding of hepatic MTX–LPD, among four cases who required additional chemotherapy after insufficient regression, three cases was negative for EBER. Therefore, EBV-positive hepatic MTX–LPD may also be a favorable sign of regression after MTX withdraw. In the current case, MTX–LPD were completely necrosis and spontaneously regressed after MTX discontinuation, despite ERER was negative.

When MTX–LPD is suspected, clinicians should consider tissue sampling, discontinuation of MTX, and monitoring for at least 2 weeks. If improvement is observed during this period, the patient’s progress will be followed up; however, if there is no change or worsening, chemotherapy should be considered [6]. Tissue sampling was recommended within 2 weeks after discontinuation of MTX, because approximately 70% of MTX–LPD was regress in 2 weeks [22]. 12–16. If spontaneous regression was observed after discontinuation of MTX, the tumor could be clinically diagnosed with MTX–LPD [22]. In those cases, tissue biopsy or additional treatment was not necessary except for recurrent or regrowth the disease [22]. Additional treatment for hepatic MTX–LPD has not been established. According to primary hepatic lymphoma, chemotherapy was a 1st line therapy, and it was crucial for tissue sampling [23, 24]. If the tumor was solitary without extrahepatic lesion, surgical intervention was considered [24]. However, these diseases generally show the good response for chemotherapy and all resected cases reporting hepatic MTX–LPD including our case, described that they perform hepatectomy for primary hepatic tumor, then the tumor was pathologically diagnosed with hepatic MTX–LPD postoperatively. Therefore, the significance of surgical intervention for solitary or oligo hepatic lesion was not unclear.

Patients with hepatic MTX–LPD were generally good, and no recurrent lesion was reported in this literature review; however, the follow-up period was short (median follow-up period was only 11 months, range 4–24 months). 5-year survival of all patients with MTX–LPD was 58.9% [1]. In the other words, this disease is sometimes recurrent and life threatening. Therefore, regular follow-ups are necessary for hepatic MTX–LPD each 3–6 months according to malignant lymphoma [23]. However, appropriate duration and interval of follow-up time was not established for MTX–LPD.

## Conclusion

MTX–LPD occurred in all lesions, including those in the liver. Many patients show spontaneous regression after the withdrawal of MTX. Therefore, clinicians should consider the possibility of this rare form of liver disease. Liver masses detected in patients who had been treated for RA using MTX should be considered for tumor biopsy.

## Data Availability

All data generated or analyzed during this study are included in the published article.

## Abbreviations

MTX: Methotrexate
RA: Rheumatoid arthritis
LPD: Lymphoproliferative disease
MTX–PLD: Methotrexate-associated lymphoproliferative disease
CT: Computed tomography
CRP: C-reactive protein
MRI: Magnetic resonance imaging
EBV: Epstein–Barr virus
EBRB: EBV-encoded small RNA
FDG-PET/CT: 18F-fluorodeoxyglucose positron emission tomography CT
